# The effectiveness of integrated care interventions in improving patient quality of life (QoL) for patients with chronic conditions. An overview of the systematic review evidence

**DOI:** 10.1186/s12955-017-0765-y

**Published:** 2017-09-29

**Authors:** Sarah Flanagan, Sarah Damery, Gill Combes

**Affiliations:** 1 Research Fellow, Institute of Applied Health Research, College of Medical and Dental Sciences, University of Birmingham, Edgbaston, West Midlands B15 2TT UK; 2 Collaboration for Leadership in Applied Health Research and Care (CLAHRC) West Midlands Research Lead for Chronic Conditions Theme, Institute of Applied Health Research, College of Medical and Dental Sciences, University of Birmingham, Edgbaston, West Midlands B15 2TT UK

**Keywords:** Integrated care, Chronic conditions, QoL

## Abstract

**Objective:**

To determine the effectiveness of integrated care interventions in improving the Quality of Life (QoL) for patients with chronic conditions.

**Design:**

A review of the systematic reviews evidence (umbrella review).

**Data sources:**

Medline, Embase, ASSIA, PsychINFO, HMIC, CINAHL, Cochrane Library (including HTA database), DARE, and Cochrane Database of Systematic Reviews), EPPI-Centre, TRIP and Health Economics Evaluations databases. Reference lists of included reviews were searched for additional references not returned by electronic searches.

**Review methods:**

English language systematic reviews or meta-analyses published since 2000 that assessed the effectiveness of interventions in improving the QoL of patients with chronic conditions. Two reviewers independently assessed reviews for eligibility, extracted data, and assessed the quality of included studies.

**Results:**

A total of 41 reviews assessed QoL. Twenty one reviews presented quantitative data, 17 reviews were narrative and three were reviews of reviews. The intervention categories included case management, Chronic care model (CCM), discharge management, multidisciplinary teams (MDT), complex interventions, primary vs. secondary care follow-up, and self-management.

**Conclusions:**

Taken together, the 41 reviews that assessed QoL provided a mixed picture of the effectiveness of integrated care interventions. Case management interventions showed some positive findings as did CCM interventions, although these interventions were more likely to be effective when they included a greater number of components. Discharge management interventions appeared to be particularly successful for patients with heart failure. MDT and self-management interventions showed a mixed picture. In general terms, interventions were typically more effective in improving condition-specific QoL rather than global QoL. This review provided the first overview of international evidence for the effectiveness of integrated care interventions for improving the QoL for patients with chronic conditions.

**Electronic supplementary material:**

The online version of this article (10.1186/s12955-017-0765-y) contains supplementary material, which is available to authorized users.

## Background

More than 15 million people in the UK are living with a chronic condition [1] and analysis of individual conditions suggests that this number is growing. It is predicted that by 2018, health and social care services may require £5 billion in additional expenditures to provide care and treatment for this population [2]. Furthermore, patients with chronic conditions often experience fragmented care [3] and over recent years, there have been a number of service and policy-driven interventions to improve service integration for these patients [4–6].

Integration is a contested concept and the related literature features a wide range of definitions which complicates efforts to evaluate services aimed at improving integration for these patients. In this article we define integrated care as an organising principle for healthcare delivery which aims to improve patient care through better coordination of services provided [7].

Patients with chronic conditions generally experience lower QoL which can lead to negative impacts upon aspects of their life [8]; for example poor employment and worsening health outcomes [9]. QoL, like the concept of integration, has a variety of definitions and includes a range of aspects pertaining to lived experience, although it can be encapsulated as a ‘broad multidimensional concept that usually includes subjective evaluations of both positive and negative aspects of life’ [10]. Measuring QoL helps to capture the ‘personal and social context’ of a patients’ experience of health [11]. Although health is a prominent aspect of QoL, there are other social determinants of heath including domains such as social interactions, work and housing.

The measurement of patient QoL is increasingly important to both the National Health Service (NHS) and social care [12]. Formal assessment of QoL is now a mandatory requirement in most clinical trials where QoL is considered an important outcome measure [13].

Whilst enhancing QoL for people with chronic conditions is important on a patient level, improving QoL is also likely to (albeit indirectly) address some of the challenges facing the NHS, including increased service demand with a growing population, and treating an aging population in a system beset with budget cuts and limited resources. Enhancing QoL for patients with chronic conditions has been identified as a priority area for the NHS [12].

Measuring patients’ QoL and health outcomes on a routine basis has a number of potential benefits. It can provide evidence to inform the professional regulation of clinicians based on health gains in patients along with the performance of hospitals and clinical teams providing care at all stages of patient care. Furthermore, it can inform decisions about the most appropriate information to give to patients to enable them to make informed choices about their treatment options, care, and management of their condition. It could also have fiscal implications as QoL measurements could provide evidence to inform decisions about where money spent would produce the best results [14].

This paper outlines the findings of an umbrella review synthesising review-level evidence that assessed the effectiveness of integrated care interventions in improving patient QoL for patients with chronic conditions. The results reported compliment the findings from a larger study [15] in which QoL was one of a number of outcomes of interest. Following an outline of the review methodology, the results will be presented giving an overview of the reviews that reported QoL as an outcome, alongside an indication of the effectiveness of specific interventions for patients with chronic conditions and a description of the intervention components associated with improved QoL. The discussion and conclusion will summarise the findings and place them in the context of the current policy and research landscape.

## Method

### Selection criteria

This umbrella review aimed to identify all eligible systematic reviews of integrated care interventions published during or after 2000, written in English and pertaining to adults undergoing management of one or more chronic conditions [full methods reported elsewhere - Damery et al. 2016]. The year 2000 was chosen following scoping searches undertaken that found no systematic review evidence around integrated care prior to this year. Interventions must have been implemented in at least two health and/or social care settings (e.g. primary care, secondary care, community settings). The community setting category could encompass care given in the community, in a patient’s home, or by social care professionals. Chronic conditions were defined according to a list of 11 specific conditions that formed the basis of the most recent Health Survey for England in 2013 which included hypertension, depression, diabetes, coronary heart condition, stroke, transient ischaemic attack (TIA), chronic obstructive pulmonary condition (COPD), cancer, heart failure, dementia and arthritis. The overview study included evidence related both to fully integrated service models for the management of patients with chronic conditions and individual interventions that may form the building blocks of an integrated health and social care model.

### Outcome measures

Following a scoping exercise and consultation with patients and service providers, patient QoL was identified as one of the outcomes of interest and of importance to consider in terms of interventions to improve integration for patients with chronic conditions. Other outcomes included health and social care resource use (including hospital admission/readmission rates, length of stay, emergency department visits); health and social care costs; patient satisfaction and any measure of care co-ordination. This paper reports the findings in relation to the impact of integrated care interventions on patient QoL.

### Identification and selection of relevant studies

Full searches of the literature were conducted using the following databases: Medline, Embase, Applied Social Sciences Index and Abstracts, PsycINFO, Health Management Information Consortium database, CINAHL, Cochrane Library, EPPI-Centre Library, TRIP database and Health Economics Evaluations Database (HEED). Web-based searches were also undertaken along with reference checking and a search of PROSPERO.

Two authors (SD and SF) independently screened titles and abstracts against an pre-established inclusion/exclusion proforma and potentially eligible full texts (open access and paid for)were retrieved and assessed. Once the list of relevant reviews was agreed upon and full copies of the manuscripts had been obtained, data on the population characteristics, interventions being assessed and the outcomes of interest to the overview study were extracted from each eligible review using a predefined data extraction sheet. One reviewer undertook the data extraction, which was checked against the original manuscript by a second reviewer. All disagreements were resolved through discussion or through arbitration from a third reviewer.

### Data extraction

Data on review characteristics (databases searched, geographical scope, healthcare settings and specific condition(s) of focus), methodology (aim, research questions, number of studies included, review type), study participants, interventions, and outcomes of interest were extracted from each included review and cross-checked by SD and SF according to a predefined data extraction sheet.

### Quality assessment

The methodological quality of the included reviews was appraised independently by two reviewers (SD and SF) using a critical appraisal tool for systematic reviews based on Centre for Evidence-Based Medicine (CEBM) recommendations (5 – high quality; 0 - poor quality). Following this process, only studies that scored 3 or above were included in the final total, as there was a considerable difference between the quality of studies and amount of extractable data between studies scoring 2.5 and below and those scoring 3 and above. As before, any discrepancies in quality assessment were resolved through discussion, followed by arbitration by a third reviewer if consensus could not be reached.

## Results

A total of 41 systematic reviews assessed QoL (Additional file 1: Table S1). Twenty one reviews presented quantitative data [16–36], 17 reviews were narrative [37–53], and three were reviews of reviews [54–56]. A total of 1062 primary studies were included in the reviews (range 4 to 153). Sixteen studies did not report the number of patients included in the study, but across all 25 that did, all but four included 1000 patients or more (total 159,134; range 857 to 78,590). Studies within reviews varied in duration from one month to 60 months, with most lasting at least 12 months, although eight reviews did not specify follow up duration. All reviews were published between 2004 and 2015. Ten were published in Canada, nine in the USA, eight in the Netherlands, seven in the UK, three in Ireland and one each in Japan, Switzerland, Sweden and China.

Interventions were categorised into groups following the Cochrane Effective Practice and Organisation of Care (EPOC) taxonomy [57] and the review authors’ description of the intervention(s) they assessed. Seven broad categories of intervention were delineated: these were case management, CCM [58], discharge management, multidisciplinary teams, complex interventions, primary vs. secondary care follow-up, and self-management.

In terms of the chronic conditions being studied, 13 reviews included patients with chronic conditions [20, 24, 27, 37–40, 43, 48, 49, 51, 53, 54], 10 focused on patients with COPD [17, 19, 21–23, 26, 36, 41, 42, 47], nine included heart failure patients [28–30, 32, 34, 35, 46, 52, 56], three included stroke patients [16, 45, 50], two assessed stroke and cardiac patients [43, 44], and one review each covered mental health [25], rheumatoid arthritis [33], heart failure/COPD [56] and cancer [31] respectively. The strength of effectiveness for the intervention in each review was assessed on the basis of QoL outcomes (Table 1). In most reviews, the comparator was clinical care, although in many cases, a detailed description of usual care was not provided. Table 2 provides the key for interpreting the direction of the effect﻿.

**Table 1:** Intervention categories and QoL outcomes

Intervention category	↑	↓	↔	=	Total
Case management (CM)	2	0	2	2	6
Chronic Care Model (CCM)	6	0	4	0	10
Discharge management (DM)	4	0	1	3	8
Multidisciplinary teams (MDT)	3	0	7	0	10
Complex interventions	0	0	0	1	1
Primary vs. secondary care follow-up	0	0	1	0	1
Self-management	1	0	3	1	5
Total	16	0	18	7	41

**Table 2:** Key for Tables 1 and 3 Interpreting the direction of effect in included reviews

Symbol	Interpretation
↑	At least half of a given review’s included primary studies showed a statistically significant *increase* in a particular outcome following the intervention (or pooled results from a meta-analysis indicated a statistically significant positive association)
↓	At least half of a given review’s included primary studies showed a statistically significant *decrease* in a particular outcome following the intervention (or pooled results from a meta-analysis indicated a statistically significant negative association)
↔	A review reported mixed findings i.e. some primary studies may have shown a significant difference between intervention and control groups whereas others showed no significant differences
=	A review where no significant differences between intervention and control groups were reported in any of the included primary studies

### Quality of included studies

In terms of quality, 27 of the studies scored 4 or 5 with the remaining studies scoring 3 or 3.5. The quality criterion for which the largest number of reviews failed to score a point related to whether a valid consideration of bias across primary studies had been undertaken.

Table 3 shows the direction of effect for the 41 reviews that assessed QoL outcomes, broken down by condition rather than intervention.

**Table 3:** QoL outcomes by condition

Condition	↑	↓	↔	=	Total^a^
Chronic condition	5	0	9	0	13
COPD^a^	6	0	3	2	11
Heart failure^a^	4	0	4	2	10
Stroke	0	0	1	1	2
Stroke/cardiac	0	0	0	2	2
Mental health	1	0	0	0	1
Cancer	0	0	1	0	1
Rheumatoid arthritis	1	0	0	0	1
Total	17	0	18	7	41

^a^The review by Ontario HTA [34] included QoL data for both heart failure and COPD patients; these are displayed separately in the table, totalling 41 studies

The range of QoL measurement tools used within included reviews i presented in Table 4.

**Table 4:** Specific QoL measurement tools cited in included reviews

QoL measure used	Number of reviews
St George’s Respiratory Questionnaire	5
Short Form Heath Survey - 36	3
Chronic Respiratory Condition Questionnaire	2
The Minnesota Living with Heart Failure Questionnaire	2
Rheumatoid Arthritis QoL	1
EuroQol 5D	1
Nottingham Health Profile Questionnaire	1
Sickness impact Profile	1
Dartmouth co-op QoL questionnaire	1

### Effects by intervention type

Overall, 18/41 (42.5%) reviews assessing QoL reported some significant improvements in QoL. Although five of the seven intervention groups included at least one review that showed positive results, the most effective in proportional terms was the CCM, in which positive results were demonstrated in 6/10 reviews that assessed QoL. Discharge management interventions were also moderately effective, with 4/8 reviews in this group showing QoL benefits for patients receiving the intervention.

The summary of results will include descriptions of components of the intervention. Effect sizes are reported where they are observed in the review. However, effect sizes were infrequently reported.

### Case management

Case management interventions were based on the implementation of a collaborative process between one or more care co-ordinators or case managers and the patient, to assess, plan and facilitate service delivery for patients with chronic conditions, particularly across health and/or social care settings.

Six reviews focused on case management interventions: three for patients with chronic conditions [37–39], one for stroke patients [16], one for patients with COPD [17], and one study focused upon patients with heart failure [18]. Two reported positive findings [18, 39], two reported mixed results [37, 38], and two reported no differences between intervention and control groups across the primary studies they included [16, 17].

The two positive reviews were both of moderate quality (Quality assessment (QA) score 3)) although neither of these reviews presented any effect sizes for QoL estimates made, rather, giving an overview of these positive findings. The first review focused on patients with chronic conditions [39], and the other included patients with heart failure [18]. In the first of these studies [39], care planning and co-ordination were undertaken by patient navigators acting as advocates for patients to smooth the transition between care providers. Care planning was delivered in a variety of ways - via the telephone, home visits, liaison with medical and community services, and/or patient education. The authors noted that the evidence suggests that the intervention should start prior to discharge, possibly as early as or just after admission. The second review [18], categorised interventions according to the intensity of patient contact provided. For example, intensive follow up entailed a face-to-face appointment every 4 to 6 weeks for up to 18 months, decreasing with intensity and follow ups were determined by patient need.

### Chronic care model

CCM interventions focused on the use of Wagner et al.’s CCM for chronic condition management that identifies elements of the health care system that can facilitate high quality [58]. Interventions in this category were based on assessing the effectiveness of one or more chronic care components (community, health system, self-management support, delivery system design, decision support, and the use of clinical information systems) in improving outcomes.

Ten reviews assessed interventions based on the CCM. Six reviews focused on patients with COPD [19, 21–23, 41, 42]. Three reviews focused upon patients with chronic condition [24, 40, 54,], and one review included patients with mental health problems [25].

Six reported positive findings with respect to QoL outcomes [21, 24, 25, 41, 42, 54], and four reported mixed results [19, 22, 23, 40] with three of the positive reviews focused on patients with COPD, two on chronic condition and one on mental health.

Kruis et al.’s (2013) high-quality review [21] (QA 5) included interventions focused on the provision of integrated condition management for patients with COPD. Interventions were grouped according to the particular emphasis/combinations of components that were described. These components included: exercise, self-management, structured nurse/General Practitioner (GP) follow-up, educational sessions, and individually tailored education. Integrated condition management showed a statistically and clinically significant improvement in conditions specific QoL on all domains of the Chronic Respiratory Questionnaire [59]. The CCM interventions included in Steuten et al. [42] included self-management and delivery system design components, with several programmes also including decision support and/or clinical information systems components. The authors reported data for 23 primary studies and the results were more positive in the studies reporting condition-specific measures. Thirteen of the 23 primary studies used the St George’s Respiratory Questionnaire (SGRQ) [60], showing a mean difference on total scores of −3.71 in favour of the intervention group (95% CI: -5.83 to −1.59, *p* < 0.0001) at up to 12 months follow up. Two further primary studies measured QoL using the SGRQ at 18 or 24 months and found no differences between intervention and control groups (*p* = 0.95). Eight primary studies used the Chronic Respiratory Condition Questionnaire. Each domain on the assessment measure showed statistically significant differences in favour of the intervention at 12 months. Two primary studies that used the same measure at 24 months did not show the same results – one found no difference between groups, whereas the other found a significant positive result on a single domain of the assessment tool.

The third positive review focused upon COPD, Niesink et al. [41] with interventions designed to integrate inputs, delivery, management and organisation of services for diagnosis, treatment, care, rehabilitation, and health promotion for patients. All intervention programmes included education, and exercise training, delivered by physicians, nurses, occupational therapists, psychologists, and/or dieticians through scheduled appointments in which patients were regularly monitored, and offered psychological support, relaxation therapy, smoking cessation advice, and breathing retraining. Five out of 10 studies showed statistically significant positive outcomes on one or more of the domains of the QoL instrument used. All chronic condition management programmes located in primary care improved QoL.

Two further CCM reviews reporting positive results focused upon management of patients with chronic condition. Interventions described in Hisahige [54] all had more than one component of the CCM, and were typically multidisciplinary approaches, with clinical follow up provided by specialists, home visits, hospital discharge planning or post-discharge follow-up, counselling in hospital, and patient education or reminders. Over half (12/21) of included reviews assessing QoL observed improvements. Interventions described in Tsai et al. [24] included self-management support and/or delivery design elements, usually in combination with at least one other element, which differed across primary studies. The authors reported both overall QoL and QoL by condition sub-group. Pooled overall QoL was reported in 24 primary studies, showing the intervention to be associated with significantly improved QoL (RR 0.11, 95% CI: 0.02 to 0.21, *p* = 0.023). Sub-group analysis by condition showed no effect on QoL for asthma (12 primary studies) or diabetes (3 primary studies), but statistically significant benefits in favour of the intervention were evident in patients with depression (three primary studies) and heart failure (six primary studies).

The final positive review Woltmann et al. [25] (QA score 5), focused on patients with mental health problems and included interventions with at least three components of the CCM. Six primary studies that reported mental health QoL were pooled and showed a significant improvement in the intervention group (Cohen’s d = 0.20, 95% CI: 0.04 to 0.36). Similarly, physical health QoL (six primary studies) also showed a significant improvement in favour of the intervention (Cohen’s d = 0.33, 95% CI: 0.17 to 0.49).

### Discharge management

Discharge management (DM) interventions were designed to facilitate effective transition from hospital care to another setting and interventions typically included a pre-discharge phase of support, transitional support and post-discharge follow-up.

Eight reviews in the discharge management group assessed QoL outcomes: three of these for patients with heart failure [28, 30, 46,], one for stroke patients [45], one for patients with chronic conditions [27], one for COPD patients [26], and two for patients with both stroke and cardiac conditions [43, 44]. All three heart failure reviews reported positive results, as did the review on chronic condition management [27]. Three further reviews found that discharge management interventions were inconclusive in terms of improving patient QoL [26, 43, 44].

Of the five positive reviews, Philips et al. [28] included interventions offering post-discharge support for patients hospitalised with heart failure. The kind of support was categorised as a single home visit in which heart failure education and self-care were reviewed and reinforced; a regime of increased clinic follow up and/or frequent telephone contact for education, self-care and to reschedule missed clinic appointments; extended multidisciplinary home care services; or day hospital services. Only overall QoL was provided by the authors, who reported that in six primary studies assessing QoL, there was a greater improvement compared with baseline in the intervention group compared to control patients (25.7% vs. 13.5%, *p* = 0.01).

Phillips et al. [29] included interventions focused on the implementation of specialist nurse-led clinics for heart failure patients. The interventions were grouped into ‘complex’ interventions which included both hospital discharge planning and post-discharge follow-up, and ‘less complex’ interventions which incorporated fewer discharge management components. QoL analysis by sub-group was not possible due to heterogeneity across primary studies, so the authors simply reported overall QoL across five primary studies. Most studies demonstrated that QoL scores improved relative to baseline scores. There was a trend towards a greater percentage improvement in QoL scores for intervention patients than controls, although this was not statistically significant (30.6 +/− 20.7% vs. 19.3 +/− 12.6%, *p* = 0.13).

The third positive review targeted patients with heart failure [47]. The authors assessed the effectiveness of post-discharge interventions delivered via home visits; heart failure clinic visits and/or telephone support; multidisciplinary care and case management to provide structured discharge planning; and patient education and self-care management. The interventions showed promising effects in improving both QoL and the functional status of patients. The final positive review included patients with general chronic condition [27], and evaluated interventions that comprised either discharge planning or comprehensive discharge planning with post-discharge support. Five of the studies included in this review assessed discharge planning alone and found the intervention to be more effective than usual care. The remaining six included studies focused upon discharge planning plus post-discharge and the authors reported that these showed even greater improvement in general QoL (effect sizes were not provided).

### Multidisciplinary teams

MDT interventions all featured the use of multidisciplinary teams as a substantive component, even when the intervention was combined with other features (e.g. post-discharge support, self-management advice, or case management).

Ten reviews assessed the effectiveness of interventions in which MDT care was the substantive component. Four of the reviews were for people with heart failure [32, 34, 36, 56], three for patients with chronic conditions [43, 48, 49], one for patients with cancer [31], one for patients with rheumatoid arthritis [33] and one review for patients with COPD [47]. Three of these reviews reported positive findings for QoL [33, 47, 48]. The remaining seven reviews reported mixed findings [31, 32, 34, 35, 43, 49, 56].

Of the three positive reviews, Ndosi et al. [33] evaluated interventions that included specialist nurses, nurse practitioners, or other nurses practicing at an extended role. This could be either in the form of supplementation (nurse working alongside physicians) or substitution (nurses performing the role that would otherwise be undertaken by a physician). One primary study within this review assessed condition-specific QoL, and reported a ratio of means of 0.83 (Cohen’s d) between QoL measurements in intervention and control groups (95% CI: 0.75 to 0.92, *p* < 0.001), suggesting that nurse-led care can significantly improve QoL for patients with rheumatoid arthritis.

Sikich [47] included primary studies with interventions delivered for patients with COPD by a range of professionals as a team under a single organisational umbrella, or through a range of organisations brought together as a unique team. Teams included respiratory specialists and a MDT that included a physician. The content of the intervention was based on the components of the CCM. The effectiveness of the intervention with regard to QoL was assessed in three primary studies. In all of these primary studies, the mean change score from baseline to the end time point in the intervention group (between 3 and 12 months post-intervention) showed either improvement compared to control or less deterioration compared to control. These observed changes were statistically significant in all three primary studies (although the authors report that the quality of evidence is poor) – the pooled weighted mean difference in the total SGRQ score was −4.05 (95% CI: -6.47 to −1.63, *p* = 0.001).

Finally, Smith et al. [49] included interventions that comprised liaison meetings attended by specialists and a patient’s primary care team to discuss and plan ongoing patient management. Other components of the intervention included sharing of patient records between professionals involved in patient care. Three of the included primary studies reported significant benefits in favour of the intervention, one study found no significant differences between the intervention and control, and one other study assessed changes in QoL from baseline and reported significant improvement in the intervention on the Minnesota Living with Heart Failure Score [61].

### Complex interventions

Complex interventions included a range of interventions in their assessments, such as multidisciplinary teams, case management or discharge management. One high quality review [30] (QA 5) assessed complex interventions (more than one intervention or service model) for heart failure patients. The interventions included case management, telephone and home visits, specialist nurse-led clinics, and multi-disciplinary interventions to bridge the gap between acute and home settings. However, the authors found no differences in QoL between intervention and usual care groups.

### Primary vs. secondary care follow-up

These interventions focused on the substitution of care from the standard secondary care setting to primary care, or enhanced primary care integration with secondary care providers. A single review [50] assessed interventions for stroke patients based on the provision of care through primary care services, which might otherwise have been provided by secondary care, reporting mixed results. One study indicated significant improvements in the mental health component of the SF-36 but the authors reported that this was a small study of weak quality. None of the other of the six studies showed any significant difference in measures on QoL.

### Self-management interventions

Five reviews assessed QoL following self-management interventions. One high-quality review [36] (QA 5) reported positive findings, three reviews focusing on patients with chronic conditions reported mixed results [20, 49, 51,], and one review of interventions for heart failure patients [52] reported no differences in QoL between intervention and control groups. Zwerink et al. [36] described a series of structured COPD interventions designed to improve self-management skills. Eligible primary studies needed to include at least two of the following: action planning, exercise programme, smoking cessation or dietary advice, medication management, coping with breathlessness training, cognitive behavioural therapy, motivational interviewing, goal-setting and feedback. The authors reported findings from 10 primary studies which measured QoL using the SGRQ. Across these studies, the mean SGRQ score was 3.51 points lower in the intervention group compared to controls (95% CI: -5.37 to −1.65), showing a statistically significant improvement in condition-specific QoL for patients receiving the intervention, with particular improvements demonstrated in the domains of symptoms, activity, and daily living.

## Discussion

This paper summarises the findings from a review of reviews assessing the effectiveness of integrated care interventions in improving QoL for patients with chronic conditions. Taken together, the 41 reviews that assessed QoL as an outcome provided a mixed picture of the effectiveness of integrated care interventions, although there were some key findings that point towards particular interventions that may be effective for patients in some condition areas. The key findings relating to QoL are summarised below.

Case management interventions showed some positive findings with regard to QoL. Care planning and co-ordination by patient navigators improved QoL for patients requiring general chronic condition management [39], and one-to-one case management via a specialist nurse or cardiologist significantly improved QoL for patients with heart failure [18]. Similarly, CCM interventions also showed promise, with six of the ten reviews demonstrating positive results. Three of these positive reviews focus on patients with COPD [21, 41, 42,]. In all cases, CCM interventions were more likely to be effective in improving QoL when they included a greater number of components; e.g. Woltmann et al. [25] found that interventions with three or more components significantly improved QoL for mental health (Cohen’s d = 0.20, 95% CI: 0.04 to 0.36).

Discharge management interventions appeared particularly successful in improving QoL for patients with heart failure, with three out of three reviews in this patient group demonstrating positive findings [28, 29, 46]. Discharge management for stroke patients showed mixed results, but hospital outreach and hospital-directed home rehabilitation was more likely to be associated with improved QoL than conventional community rehabilitation services.

MDT interventions showed primarily mixed results with regard to improvements in QoL. However, nurse-led care [33] was associated with a significant improvement in QoL for patients with rheumatoid arthritis.

Self-management interventions showed a mixed picture; three reviews reported mixed findings [20, 51, 53], and one review reported no differences between intervention and control groups [52] in changing QoL. However, Zwerink et al. [36] outlined a complex intervention for COPD support, training, and symptom management that showed some significant improvements in condition-specific QoL in patients receiving the intervention (St George’s Respiratory Questionnaire scores improved by 3.51 points in the intervention group compared to usual care) and Coulter et al. [20] demonstrated some improvements in health related QoL.

More complex interventions appeared to be more effective than less complex interventions e.g. McMartin [27] showed that discharge planning alone improved QoL but that discharge planning plus ongoing post-discharge support led to even greater QoL gains for patients with chronic conditions.

Specialist input into care was effective at improving patient QoL in general, as were interventions with shared care across the primary and secondary care interface [48], although it was not clear whether any particular component of the shared care intervention (liaison meetings, patient-held shared care record, or common computer access across settings) contributed more or less to observed QoL improvements than others.

In general terms, interventions were typically more effective in improving condition-specific QoL rather than global QoL [21, 47], as would be expected in patients with chronic conditions given that generic QoL measures are typically validated for use in the general population.

In reviews where significant QoL improvements were noted for patients receiving an intervention, improvements tended to be short-lived, with QoL improvements seen at 6 or 12 months follow up no longer discernible at the 18 or 24 month follow up stage (e.g. Kruis et al. [21] found a statistically significant improvement in QoL for COPD patients at 12 months – 3.71 point improvement in the SGRQ, but no differences between groups at 18/24 months period);

Although many reviews reported statistically significant positive findings, this significant improvement was often only seen on a single domain of a multi-component QoL scale such as the SF-36.

### Strengths

This overview study presents the first comprehensive review of the international evidence related to the effectiveness of integrated care interventions in improving QoL of patients with long-term conditions. As the review included systematic review evidence rather than a review of primary studies, we were able to assess a large volume of evidence across diverse conditions, interventions and settings. This method of assimilating a large volume of evidence and presenting the findings in a broader sense increases its potential value to commissioners and care providers. A further strength of the review was the thorough search methods employed to retrieve the evidence, assess its eligibility, extract the relevant data and assess the quality of the reviews included.

### Limitations

Where interventions were associated with a significant improvement in QoL, figures regarding the magnitude of the improvement were typically not provided by review authors, so it is difficult to assess the relative effectiveness of different interventions in leading to QoL increases. The authors of many reviews that assessed QoL outcomes found that the data in the primary studies was of poor quality. In general their findings reflected an aggregation of low quality or weak evidence, so the QoL findings presented in this paper must be interpreted with caution. That said, this is more a limitation of the studies included in the reviews than of our review of reviews. Where statistically significant findings were reported in a review, this was often on the basis of only two or three (usually poor quality) primary studies which had reported QoL improvements. In many reviews, specific QoL tools were not named, and QoL assessments were often undertaken pre- and post-intervention and not compared to a control group that did not receive the intervention. Although we tried to group interventions as separately as possible, there were also a number of overlaps between intervention. This meant it was not always possible to ascertain which particular aspects of an intervention were most effective. Commonly, specific interventions included components from a range of different interventions. For example, CCM interventions usually included a self-management component and discharge management interventions were often delivered through multidisciplinary teams. This needs to be borne in mind when interpreting the findings.

### Research and policy implications

QoL improvements often appeared to be short lived, with QoL gains made within the first 12 months following an intervention typically not persisting to the 18 or 24 month stage (e.g. Kruis et al. [21]). This illustrates a wider point about the extent to which evaluations of the effectiveness of integrated care interventions are made at the most timely points, given that different outcomes may show results at different rates. For example, hospital admission or readmission outcomes tend to be known early on in the timescale of an intervention and its post-implementation follow up period. Other factors such as QoL and patient satisfaction may take longer to become apparent, but post-intervention follow up periods are often too short to fully capture the extent to which these improvements may persist over time. This can make it difficult for policy makers to make recommendations based upon short-scale outcomes.

Furthermore, reviews included in the overview study described a wide range of measures for assessing QoL. This diversity of measures and ratings scales meant that synthesising evidence for this outcome was impossible in anything but a qualitative way. Some standardisation of assessment tools to measure patient QoL is required so that robust assessment of the evidence can be undertaken.

Most of the reviews included focused on patients with a single chronic condition rather than patients with multimorbidities. Patients with multimorbidities often face unique challenges when navigating multiple care providers, and their QoL can be further compromised whilst facing these additional challenges. Future work should focus upon patients with multimorbidities and developing interventions to promote effective, coordinated care for these patients should be a priority.

The context in which interventions are implemented is also often overlooked. In other words, what seems to work in one setting may not work in another setting. As noted, there are also particular specific condition areas that are most likely to benefit from integration e.g. COPD and Heart Failure, but there is no universal recipe or toolkit for effective interventions.

The evidence for effectiveness was mixed, although there were some promising findings indicating that some interventions may improve patient QoL. In general, multi-component, condition-specific interventions were more likely to lead to positive patient outcomes, and these findings echo those presented by Damery S et al. [15] that highlighted effective interventions to reduce hospital use for patients with chronic conditions.

## Conclusion

This review provides the first overview of the international evidence for the effectiveness of integrated care interventions for improving the QoL for patients with chronic conditions. Improved patient QoL is a goal for policy makers and health and social care providers alike and improvements should lead to positive impacts upon patient satisfaction, lower service utilisation and more timely resource allocation. Further work around establishing a formal system to measure patient QoL is needed, along with ensuring that the data are used to inform decisions about and improve patient care and satisfaction. It is worth noting that although the results are generally mixed, there were no observed reductions in QoL for patients as a result of undergoing an intervention.

## Additional file

Additional file 1: Table S1. Characteristics of included reviews. (DOCX 46 kb)

## Abbreviations

CCM: Chronic Care Model
CEBM: Centre for Evidence-Based Medicine
CI: Confidence Interval
CLAHRC: Collaborations for Leadership in Applied Health Research and Care
COPD: Chronic Obstructive Pulmonary Condition
CRDQ: Chronic Respiratory Disease Questionnaire
EPOC: Effective Practice and Organisation of Care
GP: General Practitioner
HEED: Health Economics Evaluations Database
MD: Multidisciplinary
MDT: Multidisciplinary team
MI: Myocardial Infarction
NHS: National Health Service
QoL: QoL
RCT: Randomised Control Trial
RR: Relative risk/Risk Ratio
TIA: Transient ischaemic attack
