# Supplementary material for: The effectiveness of integrated care interventions in improving patient quality of life (QoL) for patients with chronic conditions. An overview of the systematic review evidence
Source: Health Qual Life Outcomes. 2017 Sep 29;15:188. doi: 10.1186/s12955-017-0765-y (PMC5622519; doi:10.1186/s12955-017-0765-y)
Supplement: Additional file 1: Table S1. — Characteristics of included reviews. (DOCX 46 kb) [file 12955_2017_765_MOESM1_ESM.docx]

**Table 1** **Characteristics of included reviews**

| **Identifier** | **Study design features** | | | | | | | **Intervention** | | **QA** | **Study findings** |
| --- | --- | --- | --- | --- | --- | --- | --- | --- | --- | --- | --- |
| Author (year), country | Review type | | Study designs,  n | | Condition area(s),  No. of participants | Comparator | | Intervention characteristics | Time-scale | QA score (out of 5) | Review results |
| CASE MANAGEMENT | | | | | | | | | | | |
| 1. Ellis et al (2010), UK [16] | SR and meta-analysis | | RCTs  n=16 | | Stroke  n=4,759 | NS | | - Referral to stroke liaison workers (SLW) (health/social care or voluntary sector) - SLW providing one or more of education and information, social support, liaison with other services. - Proactive and structured: fixed number of visits before or after discharge - Reactive and flexible: to meet needs as they arose - Proactive and focused: fixed duration and consultations on specific issues like risk factors | NS | 5 | 13 studies assessed subjective health status. None found significant differences between intervention and control. Subjective health status standardised mean difference -0.03 (95% CI: -0.11 to 0.04, p=0.34). Extended activities of daily living standardised mean difference 0.04 (95% CI: -0.03 to 0.11, p=0.22).  Sub-group analysis (n=14, 3,114 participants) did not suggest any overall QoL benefit from stroke liaison worker interventions. |
| 2. Hickam et al (2013), USA [37] | Narrative | | RCTs, observational  n=153 | | Chronic condition  NS | Care without a case management component | | - Some intensive interventions, multiple face-to-face interactions and home visits - Some interventions entailed infrequent contact - Case managers working independently or with a MDT of health professionals | 6-36 mo | 4 | **Overall:** Case management was frequently successful in improving aspects of QoL directly targeted by the intervention but improvements were either small or of unclear significance.  Case management was less successful at improving overall QoL as indicated by global measures not specific to a particular condition.  **Condition specific**: 6 studies showed case management programmes for HF increased HF-related QoL (low quality).  8 studies on cancer showed no improvement in QoL (low quality). |
| 3. Latour et al (2007), Netherlands [38] | Narrative | | RCTs, controlled trials, before/after, time series  n=10 | | Chronic condition  n=5,092 | Care without a case management component | | - Post-discharge nurse-led case management for complex patients - Outpatient setting - Assessment of patient needs, developing service plan, arrangement of service delivery, monitoring and assessment, evaluation and follow-up - Interventions sometimes included home visits, others telephone follow-up only | 3-18 mo | 3 | 4 studies. 3 (one high quality study and 2 poor quality studies) showed no QoL difference between intervention and control groups. One further study reported a significant improvement in QoL in the intervention group (low quality). |
| 4. Manderson et al (2012), Canada [39] | Narrative | | RCTs  n=15 | | Chronic condition  n=2,317 | NS | | - Care planning, co-ordination - Phone support, home visits, liaison with medical and community services, education - Navigators advocate for patient and broker access to care when transitioning across settings or providers - Delivered by advanced practice nurses, care co-ordinators, case managers | 1-18 mo | 3 | 9 studies. 5 reported an increase in patient QoL on at least one of the domains measured; 4 reported no difference between intervention and control groups. |
| 5. Taylor et al (2005), UK [17] | SR and meta-analysis | | RCTs  n=9 | | COPD  n=1,428 | Conventional post-discharge care | | - Brief (1 month) or longer term (12 months) interventions - Inpatient, outpatient and community based - Led, co-ordinated or delivered by respiratory nurses - Home visits, telephone follow-up - To promote self-care or self-management, give lifestyle advice | 3-12 mo | 4.5 | 3 studies. Condition-specific QoL using the St George’s Respiratory Questionnaire showed no difference between intervention and control groups at 3-12 months follow-up. |
| 6. Thomas et al (2013), UK [18] | SR and meta-analysis | | RCTs  n=10 | | Heart failure  NS | NS | | - Condition management education one-to- one with specialist nurse or cardiologist - Varied intensity: - Intensive follow-up: 4-6 week appointments - Every 1-2 weeks for 3 months, then every 3 months - Regular follow-up: 3-4 month appointments - 4) Tailored: appointments by patient need | 3-18 mo | 3 | 5 studies. Improved QoL was noted in 4 studies; 1 study failed to show any improvement in QoL in the intervention group compared to control. |
| CHRONIC CARE MODEL/CONDITION MANAGEMENT | | | | | | | | | | | |
| 7. Adams et al (2007), USA [19] | SR and meta-analysis | | RCTs  n=32 | | COPD  NS | NS | | - Interventions contained at least 1 component of the CCM - Categorised according to number of components an intervention included | 6wks-24 mo | 4 | **Interventions with 1 CCM component**: 10 studies. Two studies (140 patients) demonstrated clinically and statistically significant improvements in QoL for the intervention; the other 8 studies (1348 patients) showed no difference between groups.  **Interventions with 2 or more CCM components**: 6 studies (1040 patients). None showed a significant difference between intervention and control in pooled analysis. |
| 8. de Bruin et al (2012), Netherlands [40] | Narrative | | RCTs, before/after, controlled trials, post-test only, case control  n=41 | | Chronic condition  n=78,590 | NS | | - Studies categorised according to the number of components from Wagner’s chronic care model they included | NS | 4.5 | 13 studies.  9 found no significant difference between intervention and control groups.  2 reported that the intervention improved mental function but not physical function domains.  1 reported an improvement in both mental and physical functioning in the intervention group.  1 reported a significant improvement in most QoL domains for the intervention.  The authors conclude that there is insufficient evidence about a positive association between intervention and QoL. |
| 9. Hisahige (2013), Japan [54] | Review of reviews | | Systematic reviews and meta-analyses  n=28 | | Chronic condition  NS | NS | | - Interventions all had more than one component of the chronic care model - Typically multidisciplinary approaches with clinical follow-up by specialists, home visits, hospital discharge planning or post-discharge follow-up, counselling in hospital and patient education or reminders | NS | 3.5 | 21 studies. 57% (12/21) reported an observed improvement in QoL with a reasonable amount of evidence. |
| 10. Kruis et al (2013), Netherlands [21] | SR and meta-analysis | | RCTs  n=26 | | COPD  n=2,997 | Regular follow-up visits to healthcare providers | | - Integrated condition management with: - Exercise dominant - Self-management dominant - Structured nurse/GP follow-up - Exercise and self-management - Self-management and structured follow-up - 6) Educational sessions and individually-tailored education | 3-24 mo | 5 | 23 studies measured QoL, using 6 different measures.  **General QoL**: 6 studies. No between-group differences found in pooled analysis for SF-36 (3 studies). Small sample sizes.  2 further studies using the Sickness Impact Profile also reported no differences between groups on any domain. One further study (Dartmouth Primary Care Cooperative QoL Questionnaire) reported significantly higher QoL in intervention group compared to baseline (no control group) at 12 months.  **Condition specific**: St George’s Respiratory Questionnaire (13 studies, 1,425 patients). Pooled mean difference on total score -3.71 in favour of intervention (95% CI: -5.83 to -1.59, p<0.001) at up to 12 months follow-up. 2 studies (189 patients) measured SGRQ at 18 or 24 months follow-up and found no difference between groups (p=0.95).  Chronic Respiratory Questionnaire (8 studies, 4 in meta-analysis, 160 patients). Each domain showed statistically significant differences in favour of intervention at 12 months. 2 studies (151 patients) measured CRQ at 24 months. One found no difference between groups; the other found a significant difference in favour of the intervention on the dyspnoea domain. |
| 11. Lemmens et al (2009), Netherlands [22] | SR and meta-analysis | | RCTs, before/after  n=36 | | COPD  NS | Care without a CCM component or with a single component | | 1) Patient education with case management  2) Patient education and case management with professional education  3) Patient education with substitution of physician by nurse  4) Professional and patient education in combination with pharmacists having active role in patient monitoring | 6wks-24 mo | 5 | **Patient education with case management**: 9 studies. 3 reported significant beneficial differences in QoL in favour of intervention.  **Patient education and CM with professional education**: 11 studies. 4 reported improvements in QoL in favour of intervention but not statistically significant.  **Patient education and revision of professional role**: 6 studies. 1 reported significant between-group differences in QoL.  **Professional and patient education in combination with pharmacist active role:** 5 studies. 4 reported improved (but non-significant) QoL in intervention group.  Pooled data showed intervention significantly improved QoL on several domains. |
| 12. Niesink et al (2007), Netherlands [41] | Narrative | | RCTs  n=10 | | COPD  n=868 | NS | | - Interventions to bring together inputs, delivery, management and organisation of services for diagnosis, treatment, care, rehabilitation and health promotion - All programmes included education, most also exercise training - Scheduled appointments to monitor, offer psychosocial support, relaxation, smoking cessation, breathing retraining - Delivered by physicians, nurses, occupational therapists, psychologists, dieticians | 12-18 mo | 3.5 | **Intervention vs. control group**: 10 studies. 5 reported statistically significant improvements in QoL in intervention groups.  **Intervention QoL baseline change compared pre-post intervention**: 10 studies. 7 found clinically significant differences between baseline and post-intervention QoL in favour of intervention. |
| 13. Peytremann-Bridevaux et al (2008), Switzerland [23] | SR and meta-analysis | | RCTs, controlled trials, before/after  n=13 | | COPD  n=8,179 | Care without a CCM component | | - Interventions with 2 or more components of CCM - Delivered by 2 or more health professionals e.g. respiratory nurse, physiotherapist, GP, practice nurse, social worker, case manager, pulmonary care physician - At least one component lasted for 12 months | 12 mo | 4 | 12 studies. Mixed results.  1 study showed significant improvements for intervention in all chest respiratory domains up to 12 months after intervention.  Other studies found significant impacts on one or two scale domains or selectively reported only positive results.  In 4 studies, interventions were not associated with any improvement in QoL. |
| 14. Steuten et al (2009), Netherlands [42] | Narrative | | Any with control group or outcome measured at 2 time points  n=20 | | COPD  NS | Care without a CCM component | | - Interventions with at least 2 components of the CCM - All had self-management and delivery system design - Several programmes additionally encompassed decision support and/or clinical information systems | 2-24 mo | 3.5 | 15 studies. 8 studies found statistically significant improvements for at least some QoL domains, although they were not always sustained in the longer term.  11 different QoL measures used across studies; inhibited pooling of results. |
| 15. Tsai et al (2005), USA [24] | SR and meta-analysis | | RCTs, controlled trials  n=112 | | Chronic condition  NS | Care without a CCM component | | - Interventions based on components from the chronic care model - Most interventions had self-management support and/or delivery system design, usually in combination with at least one other element | 12 mo | 3 | **Pooled overall QoL** (24 studies). Results across all studies showed the intervention to be significantly associated with improved QoL: RR 0.11 (95% CI: 0.02 to 0.21, p=0.023).  **QoL by condition sub-group**: Sub-group analysis by condition showed no effect on QoL for asthma (12 studies) or diabetes (3 studies); statistically significant benefits in favour of intervention were only evident in depression (3 studies) and HF (6 studies). |
| 16. Woltmann et al (2012), USA [25] | SR and meta-analysis | | RCTs  n=78 | | Mental health  NS | NS | | - Interventions comprising at least 3 components of the chronic care model | 3-36 mo | 5 | **Mental health QoL**: 6 studies. Pooled effect size showed a significant difference favouring the intervention. Cohen’s d=0.20 (95% CI: 0.04 to 0.36).  **Physical QoL**: 6 studies. Pooled effect size showed a significant difference favouring the intervention. Cohen’s d=0.33 (95% CI: 0.17 to 0.49). |
| DISCHARGE MANAGEMENT | | | | | | | | | | | |
| 17. Bettger et al (2012), USA [43] | Narrative | | RCTs, observational registries  n=44 | | Stroke, Cardiac  NS | NS | | 1) Hospital initiated support for discharge to home  2) Community based models of support  3) Chronic condition management models of care  4) Patient and family education, counselling, goal-setting  Provided by nurses, social workers, physical or occupational therapists, physicians, MDT in contact with patient | NS | 4 | The authors noted that available evidence identified that no tested interventions had been shown to consistently improve QoL after stroke or MI. |
| 18. Jeppesen et al (2012), Norway, UK, Australia [26] | SR and meta-analysis | | RCTs  n=8 | | COPD  n=870 | Standard discharge arrangements | | - Hospital at home - Visits in person from respiratory nurse supported by hospital team - Telephone support | 6 mo | 4.5 | 3 studies (332 patients). Very limited evidence of trend towards improved QoL in the intervention group. No meta-analysis undertaken due to poor quality of studies and high heterogeneity. |
| 19. McMartin (2013), Canada [27] | SR and meta-analysis | | RCTs, SR, meta-analyses  n=11 | | Chronic condition  NS | Standard discharge arrangements | | Interventions comprised:  1) Discharge planning vs. usual care or  2) Comprehensive discharge planning with post-discharge support vs. usual care where post-discharge support could include home visits, telephone follow-up | NS | 3 | **Discharge planning:** 5 studies. Very low quality evidence that discharge planning is more effective than usual care at improving HRQoL.  **Discharge planning plus post discharge support**: 6 studies. Very low quality evidence that discharge planning plus post-discharge support is more effective at improving QoL than usual care. |
| 20. Olson et al (2011), USA [44] | Narrative | | RCTs, observational registries  n=62 | | Stroke, Cardiac  NS | Care that did not comprise transition of care services to co-ordinate care across multiple providers | | 1) Hospital initiated support for discharge to home  2) Community based models of support  3) Chronic condition management models of care  4) Patient and family education, counselling, goal-setting | 0-12 mo | 3.5 | Early supported discharge with hospital-initiated support was associated with a reduction in length of stay that did not adversely impact QoL. The other intervention categories were not associated with any differences in QoL between intervention and control groups. |
| 21. Phillips et al (2004), USA [28] | SR and meta-analysis | | RCTs  n=19 | | Heart failure  NS | Standard discharge arrangements | | - Post-discharge support as:   1) Single home visit in which HF education and self-care were reviewed and reinforced  2) Increased clinic follow-up and/or frequent telephone contact for education, self-care, reschedule missed appointments  3) Extended multidisciplinary home care service  4) Day hospital service in specialist HF unit | 3-12 mo | 5 | 6 studies. There was a greater percentage improvement in QoL compared with baseline in the intervention group vs. control (25.7% vs. 13.5%, p=0.01). |
| 22. Phillips et al (2005), USA [29] | SR and meta-analysis | | RCTs  n=7 | | Heart failure  n=949 | NS | | - Specialist nurse-led clinics for heart failure - ‘Complex’ interventions: hospital discharge planning, post-discharge follow-up - ‘Less complex’: no hospital discharge planning and/or fewer components | 3-12 mo | 4 | 5 studies. Most studies demonstrate that QoL scores improved relative to baseline scores and there was a trend towards greater percentage improvements in QoL scores for intervention patients compared with controls, although not statistically significant. (30.6 +/-20.7% vs. 19.3 +/-12.6%), p=0.13.  QoL analysis by sub-group (less complex vs. more complex intervention) not possible due to small participant numbers. |
| 23. Winkel et al (2008), Denmark, Sweden [45] | Narrative | | RCTs  n=17 | | Stroke  n=1,122 | Standard discharge arrangements | | Delivered by MDTs which all included physiotherapists and occupational therapists; some also nurse, social worker, GP, other specialist (e.g. geriatrician)  1) Early supported discharge with hospital teams providing home rehabilitation after discharge  2) Early supported discharge with no direct rehabilitation from hospital teams  3) Community based rehabilitation after discharge | 1-12 mo | 4 | **Early supported discharge with home rehabilitation from hospital teams:** 6 studies. Results at 3 months follow-up from one high quality trial showed physical health component of SF-36 to be significantly improved in the intervention group. No differences found in other studies.  **Early supported discharge teams with no direct rehabilitation**: 3 studies. Mixed results. Studies with follow-up at 6 weeks, 6 months and 1 year found no difference between groups. One study found a beneficial impact on QoL at 3 months in favour of intervention.  **Community based rehabilitation after early discharge**: 2 studies of acceptable quality showed no difference between groups at least up until 7 months post-stroke. |
| 24. Yu et al (2006), Hong Kong [46] | Narrative | | RCTs  n=21 | | Heart failure  n=4,445 | NS | | - Post-discharge interventions via home visit, HF clinic visits and/or telephone - Multidisciplinary care and case management, structured discharge planning - All included patient education; most also self-care management | 3-50 mo | 4 | Effective programmes showed promising impacts in improving QoL and functional status for patients receiving the intervention. |
| COMPLEX/MULTIFACETED INTERVENTIONS | | | | | | | | | | | |
| 25. Takeda et al (2012), UK [30] | SR and meta-analysis | RCTs  n=25 | | | Heart failure  n=5,942 | NS | | - Condition management for HF - All led by professionals from secondary or tertiary care   1) Case management, telephone and home visits  2) Specialist nurse led clinics  3) MDT interventions to bridge gap between acute and home settings | 6-24 mo | 5 | 5 studies. Little difference reported between patients in the intervention arms vs. usual care. |
| MULTIDISCIPLINARY TEAMS | | | | | | | | | | | |
| 26. Bettger et al (2007), USA [55] | Review of reviews | | | SR of RCTs, time series, controlled trials, before/after  n=12 | Chronic condition  n=19,572 | Conventional post-discharge rehabilitation | | - Rehabilitation after hospitalisation in inpatient or community based settings - Provided by MDT including physicians, nurses, combinations of physical and occupational and speech and language therapists | NS | 4 | One study found that older patients who receive more intensive home-based multidisciplinary rehabilitation have greater social participation and QoL at 3 months. However, another study found less favourable outcomes for home-based interventions for stroke survivors compared with inpatient rehabilitation (no further detail given by authors). |
| 27. Giguere et al (2012), Canada [31] | SR and meta-analysis | | | RCTs, controlled trials, before/after, time series  n=51 | Cancer  NS | NS | | - Continuity of care - MD approaches e.g. case conference, shared documents, assessment of patient needs, care plan with measurable goals and identification and co-ordination of supplemental resources - Integration of care through each transition | 5 days-60 mo | 3.5 | **Global QoL**: 10 studies (2,622 patients). Median global QoL was 2.05 higher (95% CI: 0.06 lower to 2.14 higher). Very low quality evidence.  No differences between groups found when each QoL subscale was considered separately or when they were considered as a whole and compared across models of care.  The authors note the limited evidence for QoL benefits – studies were of poor quality and high heterogeneity limited pooling. |
| 28. Koshman et al (2008), Canada [32] | SR and meta-analysis | | | RCTs  n=12 | Heart failure  n=2,060 | Heart failure care without pharmacist involvement | | - Pharmacists educating patients on HF and HF medication - Self-monitoring support, medication management, facilitation of compliance - Directed care where pharmacist is key driver, or collaborative care with pharmacist part of MDT | 6-12 mo | 4 | 7 studies. No pooling of data was possible.  6 studies used condition-specific measures: 4 found no difference between groups and 2 found a significant difference in favour of intervention.  5 studies also included a generic QoL measure. 3 found no significant difference between groups, 1 found a significant difference in favour of control and one further study found significant changes in QoL in favour of intervention.    Results not described in review beyond the statement that results were mixed. |
| 29. Ndosi et al (2011), UK [33] | SR and meta-analysis | | | RCTs  n=7 | Rheumatoid Arthritis  n=431 | Care provided by a physician | | - Specialist nurses, nurse practitioners or other nurses practicing at an extended role   1) Supplementation (usual care from doctor vs. nurse working alongside doctor)  2) Substitution (nurse provides the same care a doctor would) | 12-24 mo | 4 | 1 study. Condition specific QoL, Cohen’s d 0.83 (95% CI: 0.75 to 0.92, p<0.001), statistically significant difference in favour of nurse-led care. |
| 30. Ontario HTA (2009), Canada [56] | SR and meta-analysis | | | RCTs  n=8 | Heart failure  n=2,692 | Care not provided by multiple practitioners | | - All included a team consisting of nurse and physician and/or primary care physician, one of which was specialist in HF management - Varying combinations of condition specific education, medication education, diet and lifestyle counselling, physical activity advice, self-care support, evidence based guidelines, regular follow-up - Delivered either through a direct model (clinic based programme) or indirect (telephone based, physician supervised, nurse-led) | 12 mo+ | 4 | 6 studies. 2 reported change scores on the physical and emotional subscales of the Minnesota Living with HF scale, of which 1 reported a significant change in favour of intervention from baseline to 12 months for the physical subscale.  No significant differences found for the emotional subscale.  The authors conclude that there is low quality evidence that the intervention is associated with improved QoL. |
| 31. Ontario HTA (2012), Canada [34] | Review of reviews | | | Systematic reviews and meta-analyses  n=24 | Heart failure, COPD  NS | Usual care in family practice | | - Specialised community based care: formalised links between primary and specialist care - Condition specific education, medication review, physical activity and lifestyle counselling, self-care, follow-up - Delivered by intermediate care teams including GPs, specialists, nurses, social workers, pharmacists, dieticians | NS | 3 | **HF-specific**: 6 studies. Only 1 study reported a statistically significant improvement in physical/emotional subscales at 1 year in favour of intervention.  **COPD-specific**: 2 studies. Both showed a mean change score for QoL that denoted a statistically significant improvement in the intervention (or significantly less deterioration in intervention compared to control group). |
| 32. Roccaforte et al (2005), Canada [35] | SR and meta-analysis | | | RCTs  n=33 | Heart failure  NS | Referral to family physician or home care services after discharge | | 1) MD approach: starting during hospitalisation, carried out up to 12 months post-discharge, delivered by various professionals, or  2) Intervention centred on specific health professionals e.g. HF specialist nurses or case managers, focused on particular components of care e.g. therapy adherence | 3-22 mo | 5 | 16 studies. 8 reported a statistically significant improvement in QoL score. 4 found discordant results, with QoL improving in the short term (6 months) but not in the longer term (12 months). 4 studies did not report any statistically significant differences between groups.  The authors note small sample sizes and large number of QoL tools across studies. |
| 33. Sikich (2012), Canada [47] | Narrative | | | Health Technology Assessments, SR, RCTs  n=6 | COPD  n=1,370 | Care not provided by multiple practitioners | | - Delivered by a range of professionals as a team under one organisational umbrella or range of organisations brought together as unique team - Most had respiratory specialist on the team, and MDT that included a physician - Interventions based on CCM components: condition specific education, medication review, physical activity counselling, smoking cessation, self-care, evidence based guidelines, regular follow-up | 3-12 mo | 4 | 3 studies. In all studies, the mean change score from baseline to end time point in the intervention group showed either improvement compared to control or less deterioration compared to control.  Changes were statistically significant in all 3 studies, but low grade evidence. Pooled weighted mean difference in total St George’s Respiratory Questionnaire score was -4.05 (95% CI: -6.47 to 1.63, p=0.001). Low quality evidence. |
| 34. Smith et al (2007), Republic of Ireland [48] | Narrative | | | RCTs, controlled trials, before/after, time series  n=20 | Chronic condition  NS | Care not provided by multiple practitioners | | - Liaison meetings attended by specialists and primary care team to discuss and plan ongoing patient management - Shared care record carried by the patient - Computer assisted shared care and email with data circulated between primary and secondary care | NS | 5 | 5 studies. 3 reported significant benefits in favour of the intervention. 1 found no significant differences between intervention and control groups. The remaining study assessed changes in QoL from baseline and reported a significant improvement in the intervention group for the physical domain and non-significant improvement on the emotional domain. |
| 35. Smith et al (2012), Republic of Ireland [49] | Narrative | | | RCTs, controlled trials, before/after, time series  n=6 | Chronic condition  n=2,298 | Care not provided by multiple practitioners | | - Any intervention to improve outcomes for patients with multimorbidity in primary or community care - Complex interventions, multiple components - Changes to organisation of care delivery, through case management or MDT | 2-24 mo | 5 | 4 studies. Three showed no difference between groups using any QoL measure. The fourth study showed a significant improvement in QoL in favour of the intervention. |
| PRIMARY VS. SECONDARY CARE FOLLOW-UP | | | | | | | | | | | |
| 36. Allison et al (2011), UK [50] | Narrative | | RCTs  n=9 | | Stroke  n=1,425 | Hospital based follow-up | | - Stroke support workers, care co-ordinators or case managers - Review of medication and assessment of long term disability - Provision of information and signposting to other services | 3-9 mo | 3 | 6 studies. One small study of weak quality reported a significant improvement in the mental health component of the SF-36 in favour of intervention.  No other studies showed any significant differences in QoL measures. |
| SELF-MANAGEMENT | | | | | | | | | | | |
| 37. Coulter et al  (2015), UK [20] | SR and meta-analysis | | RCTs, cluster RCTs | | Chronic conditions  N=10,856 | | Usual care or enhanced usual care | Face-to-face or telephone support to support patient behaviour change  Personalised care planning  All but three were primary care or community based (other three hospital based) | 1-12 months | 4 | 10 studies. Three pooled studies found no effect on physical component of the SF-36 or mental component.  Three studies measured generic health status, two found improvements related to personal care and one did not.  Four studies measured condition-specific health status and combined results showed no difference between intervention and control. |
| 38. Franek (2013), Canada [51] | Narrative | | RCTs, SR, meta-analyses  n=10 | | Chronic condition  n=6,074 | | Care from the usual provider | - Stanford chronic condition self-management programme - 6 weekly sessions of 2.5 hours, with groups of 10-15 participants - Conducted in community settings - Volunteer lay facilitators assist patients to make their own management choices and reach self-selected goals | 4-12 mo |  | 6 studies. 2 showed no significant differences between intervention and control on physical and mental QoL measures (very low quality).  1 study found a statistically significant improvement in favour of the intervention on the EUROQoL visual analogue scale (low quality).  3 studies that used the EQ-5D showed a non-significant trend towards benefits in favour of the intervention (low quality).  The authors note that QoL was sparsely reported and difficult to interpret collectively. |
| 39. Jovicic et al (2006), Canada [52] | Narrative | | RCTs  n=6 | | Heart failure  n=857 | | NS | - Education and limited follow-up - Patients taught to monitor condition, recognise exacerbation symptoms - Follow-up phone call, education delivered face to face or digitally - Delivered by nurses or allied health professionals | 3-12 mo | 4 | 3 studies (439 patients). Heterogeneity of QoL measures meant that results could not be pooled, but all studies consistently reported no significant improvements in QoL scores in the intervention group compared to control. |
| 40. Smith et al (2012), Republic of Ireland [49] | Narrative | | RCTs, controlled trials, before/after, time series  n=4 | | Chronic condition  n=1,109 | | Care not provided by multiple practitioners | - Any outcome to improve outcomes for patients with multimorbidity in primary or community care - Complex interventions, multiple components - Patient orientated interventions | 2-24 mo | 5 | 4 studies. 3 reported no significant differences between intervention and control groups for QoL. One study reported six psychosocial measures, two of which showed a significant improvement in QoL in the domains of self-efficacy in relation to fear of falling, and improvements in ‘control orientated strategies’. |
| 41. Zwerink et al (2014), Netherlands [36] | SR and meta-analysis | | RCTs, controlled trials  n=31 | | COPD  n=3,688 | | NS | - Structured COPD interventions to improve self-management skills - Studies had at least 2 of: action plan, exercise programme, smoking cessation, dietary advice, medication, coping with breathlessness, cognitive behavioural therapy, motivational interviewing, goal setting, feedback | 2-24 mo | 5 | 10 studies (1413 patients). Mean St George’s Respiratory Questionnaire total score was 3.51% lower in the intervention group compared to control (95% CI: -5.37 to -1.65) i.e. improved QoL for intervention group.  9/10 studies assessed specific QoL domains, with statistically significant differences favouring intervention on the domains of symptoms, activity and impact. |
